# Hand Fine Motor Skill Disability Correlates with Cognition in Patients with Moderate-to-Advanced Parkinson’s Disease

**DOI:** 10.3390/brainsci10060337

**Published:** 2020-06-02

**Authors:** Shennie Tan, Chien Tai Hong, Jia-Hung Chen, Lung Chan, Wen-Chou Chi, Chia-Feng Yen, Hua-Fang Liao, Tsan-Hon Liou, Dean Wu

**Affiliations:** 1 Department of Neurology, Shuang Ho Hospital, Taipei Medical University, New Taipei City 23561, Taiwan; shennietan@gmail.com (S.T.); chientaihong@gmail.com (C.T.H.); gary.320@hotmail.com (J.-H.C.); cjustinmd@gmail.com (L.C.); 2 Department of Neurology, School of Medicine, College of Medicine, Taipei Medical University, Taipei 23561, Taiwan; 3 Taiwan Society of International Classification of Functioning, Disability and Health, TSICF, New Taipei City 23561, Taiwan; y6312002@gmail.com (W.-C.C.); mapleyeng@mail.tcu.edu.tw (C.-F.Y.); hfliao@ntu.edu.tw (H.-F.L.); peter_liou@s.tmu.edu.tw (T.-H.L.); 4 Department of Occupational Therapy, Chung Shan Medical University, Taichung 40201, Taiwan; 5 Department of Public Health, Tzu Chi University, Hualien City 97004, Taiwan; 6 School and Graduate Institute of Physical Therapy, College of Medicine, National Taiwan University, Taipei 10617, Taiwan; 7 Department of Physical Medicine and Rehabilitation, Shuang Ho Hospital, Taipei Medical University, New Taipei City 23561, Taiwan; 8 Department of Physical Medicine and Rehabilitation, School of Medicine, College of Medicine, Taipei Medical University, Taipei 11031, Taiwan; 9 Graduate Institute of Injury Prevention and Control, College of Public Health, Taipei Medical University, Taipei 11031, Taiwan

**Keywords:** Parkinson’s disease, dementia, cognition, fine movement

## Abstract

In older individuals, hand fine motor skill disability is associated with cognitive levels. Similarly, patients with moderate-to-advanced Parkinson’s disease (PD) often have cognitive dysfunction. Here, we investigated the association between hand fine motor skill and cognitive dysfunction in patients with moderate-to-advanced PD. Moderate and advanced PD patients with and without dementia were identified from the Taiwan Data Bank of Persons with Disability. Hand fine motor capacities, namely pen holding, buttoning, and knotting, were assessed with the World Health Organization Disability Assessment Schedule 2.0. Statistical analyses were performed on Statistical Analysis System (SAS) and a *p* value of <0.05 was considered significant. In total, 3440 patients with PD were enrolled, of which 612 had dementia, exhibiting significant disability in all three tasks. After adjustments for age, sex, and PD severity, pen holding and knotting were significantly associated with PD dementia. The presence of any disability in either task was not only sensitive to the presence of dementia but also associated with cognitive disability in moderate and advanced PD patients without dementia. In conclusion, hand fine motor skill disability was associated with cognitive disability in patients with moderate-to-advanced PD. These simple hand fine motor skills may thus be applicable in screening tests for the early identification of cognitive dysfunction in patients with moderate-to-advanced PD.

## 1. Introduction

Parkinson’s disease (PD), the second most common neurodegenerative disease after Alzheimer’s disease (AD), affects approximately 0.3% of the general population and 2%–3% of individuals aged >65 years. PD development is mainly attributable to dopaminergic neuronal loss in the substantia nigra [1]. In addition to classical motor features, such as tremor, rigidity, akinesia, and postural instability, clinically significant nonmotor features noted in PD have attracted the attention of researchers in recent years [2]. The complexity of PD is linked to the extensive involvement of the nervous system, including both dopaminergic and nondopaminergic deficiencies [3].

The advancements in medicine and medical technologies have aided most patients with PD in remaining ambulatory independent for a longer time, throughout the PD course. However, motor disturbance is only part of the picture in patients with PD. Patients also experience nonmotor symptoms (NMSs), which adversely affect their cognition, behavior, sleep, and autonomic and sensory function [4]. Although NMSs are not recognizable as easily as motor symptoms, they can sometimes be more debilitating. Patients with PD commonly have mild cognitive impairment, with 60%–80% of them eventually developing PD dementia (PDD) [5,6]. Patients with PD and their caregivers frequently report cognitive decline as one of their greatest concerns [7,8]. In addition, PDD results in greater functional disability [9]. Therefore, the identification of PDD occurrence and its progression markers is essential.

Numerous studies have reported an association between hand fine motor skill disability and cognitive dysfunction severity [10,11,12]. Unlike AD, where memory impairment is the core symptom of dementia, the cognitive dysfunction profile in PD is heterogeneous, influencing various domains of executive function, spatial cognition, psychomotor speed, and memory [13,14]. The pathologic hallmark of dopamine neuron depletion could cause typical motor impairment in patients with PD; moreover, extensive cortical degeneration might lead to cognitive impairment in patients with PD [15,16]. Although fine motor skills are commonly impaired in neurodegenerative disorders, disease-specific changes could be observed [17]. Studies have adopted diverse tasks (finger tapping, clothes changing, pins, and pegboards) for identifying differences in fine motor skills among these patients [17,18].

Considering the dysfunction of both the motor and cognitive capacity in patients with moderate-to-advanced PD, the etiology of hand fine motor skill disability in these patients is complex. In the present study, we elucidated the association of hand fine motor skills (pen holding, buttoning and knotting) with cognitive dysfunction according to the World Health Organization Disability Assessment Schedule 2.0 (WHODAS 2.0) so as to identify the more cognition-specific hand fine motor tasks in patients with moderate-to-advanced PD.

## 2. Methods

### 2.1. Participants and Data Collection

In Taiwan, patients that fulfill the criteria of PD with modified Hoehn and Yahr (H & Y) stage 3–5 diagnosed from board-certified neurologists are eligible for disability certification and corresponding benefits from the Ministry of Health and Welfare; the claims data of these patients are recorded from the Taiwan Data Bank of Persons with Disability. Applicants with PD were selected from the database via the International Classification of Diseases (ICD), 9th Revision, Clinical Modification (ICD-9-CM) and ICD 10th Revision, Clinical Modification (ICD-10-CM) diagnosis codes ICD-9-CM 332 and ICD-10-CM G20. These may include all persons who are eligible for the first time (first time reaching the disease statue of H & Y stages 3–5) and those who are extending their disability certification. Exclusion criteria were those with secondary Parkinsonism (ICD-9-CM 332.1), omitted or missing data regarding their education level, any domain 1-6 of Functioning Disability Evaluation Scale Adult Version (FUNDES-Adult) or domain 8, and those who refused to answer. Finally, we identified and included patients with moderate-to-advanced PD who fulfilled the selection criteria and were enrolled in the aforementioned database over July 2012 to October 2018 (Supplementary Material Figure S1).

WHODAS 2.0 is a widely used measure of health and disability, covering six domains of functioning, including: Cognition (understanding and communicating, domain 1), mobility (moving and getting around, domain 2), self-care (hygiene, dressing, eating, and staying alone, domain 3), getting along (interacting with other people, domain 4), life activities (domestic responsibilities, leisure, work, and school, domain 5), and participation (joining in community activities, domain 6) [19]. On the basis of the International Classification of Functioning, Disability, and Health and WHODAS 2.0, the Functioning Disability Evaluation Scale Adult Version (FUNDES-Adult) was developed in Taiwan and added to domains 7 (environmental attributes) and 8 (motor action, capability, and capacity scores) for assessing the activity and participation norms in the Chinese culture (Supplementary Material Table S1). Each domain consists of 4–8 questions with a 5-point scale to assess the level of activity difficulty (0 = no difficulty, 1 = mild difficulty, 2 = moderate difficulty, 3 = severe difficulty, 4 = extreme difficulty). For example, for the WHODAS domain 1, which contains 5 questions (concentrating on something for 10 min, remembering to do important things, analyzing and finding solutions to problems in day-to-day life, learning a new task, understanding what people say, and starting and maintaining a conversation), the standardized scores, which range from 0 (best performance) to 20 (worst performance), are summed to obtain the total score. The absolute score in each domain is transformed to a standardized score and ranges between 0 and 100, with higher scores indicating greater difficulty. In the cognitive disability scales’ quantification, the scales are on a 10-point scale category. The questionnaires were administered to the participants or their caregivers if the participants could not answer the questions themselves.

Domain 8.1 (pen holding), domain 8.2 (buttoning), and domain 8.3 (knotting) of the FUNDES-Adult were used to evaluate hand fine motor capacity. These hand fine motor capacities were evaluated by qualified professionals. Motor activity qualifier was defined based on whether there was total independence or a need for assistance when accomplishing tasks (0 = total independence, 1 = need for supervision or mild assistance, 2 = need for moderate assistance, 3 = need for maximal assistance, 4 = need for total assistance).

The study was approved by TMU-JIRB (N201805048, approved on 4 June 2019).

### 2.2. Statistical Analysis

Statistical analyses were performed using Statistical Analysis System (version 9.2; SAS Institute Inc., Cary, NC, USA). The chi-square test was used to analyze the two categorical variables, and the Wilcoxon test was used to analyze the continuous variables. The multivariable logistic regression model was conducted to investigate the odds of dementia in PD patients with hand fine motor skill disability. In the multivariable logistic regression model, the variables included age (categorized into 4 groups as 18-64/65-74/75-84/85 and above), gender, H & Y stage (3/4/5), and the presence of any level of hand fine motor skill disability in each task (pen holding, buttoning, and knotting). Data were considered statistically significant at *p* < 0.05.

## 3. Results

We enrolled 3440 patients with moderate-to-advanced PD from July 2012 to October 2018; of them, 612 had PDD, based on simultaneous diagnosis claims in the registry. There were no significant differences in sex distribution, or secondary or higher-level education levels between the PDD and non-PDD groups; however, patients in the PDD group were significantly older. The H&Y stages and overall disability assessed using WHODAS 2.0 were significantly higher in the PDD group than in the non-PDD group. Regarding all three hand fine motor skills, including pen holding (domain 8.1), buttoning (domain 8.2), and knotting (domain 8.3), the PDD group exhibited significantly greater disability than the non-PDD group (Table 1).

**Figure 1:**
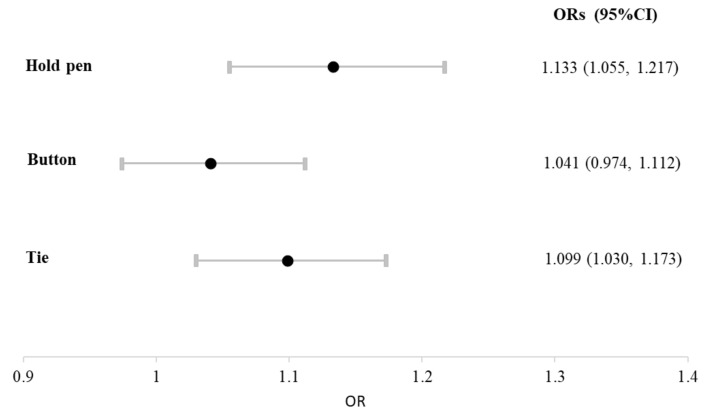
Association of disability in three hand fine motor skills with the presence of dementia in patients with moderate-to-advanced Parkinson’s disease (PD). After adjustments for age, sex, and PD severity by using multivariate logistic regression, dementia was significantly associated with disability in pen holding and knotting but not buttoning.

Because differences in age, sex, and H & Y stage could confound the association between hand fine motor skill disability and cognition, a multivariate logistic regression analysis was performed to further understand cognition-specific hand fine motor skill disability in patients with moderate-to-advanced PD. After adjustments of for age, sex, and PD severity (H & Y stage), the presence of any disability in pen holding was significantly associated with dementia (odds ratio [OR]: 1.13, 95% confidence index [CI]: 1.055–1.217), which indicated a 13% higher probability of dementia for PD patients if they had any level of disability in pen holding. The association was significant in knotting (OR: 1.099, 95% CI: 1.030–1.173) as well but not in buttoning (OR: 1.041, 95% CI: 0.974–1.112; Figure 1 and Supplementary Material Table S2).

Considering pen holding and knotting performance was significantly associated with dementia in patients with moderate-to-advanced PD, we further investigated whether the presence of any disability in either one of the two aforementioned tasks was sensitive to the presence of dementia. The incidence of any disability was significantly higher in the PDD group than in the non-PDD group (*p* = 0.004; Table 2). In addition, setting the disability of the two hand fine motor skills as the stratification parameter for dementia in patients with moderate-to-advanced PD led to sensitivity levels of up to 72.7%. Furthermore, the sensitivity reached 76.5% in patients aged ≥75 years (Supplementary Material Table S3).

**Figure 2:**
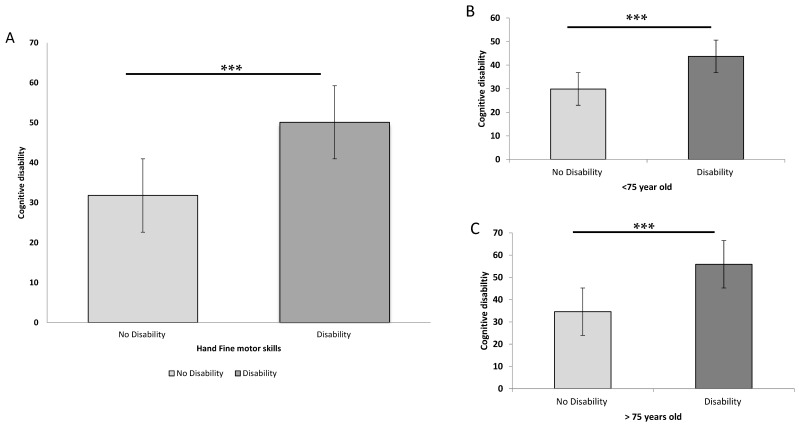
Comparison of cognitive disability between moderate-to-advanced Parkinson’s disease (PD) patients without dementia but with hand fine motor skill disability and those without dementia or hand fine motor skill disability. (**A**) Cognitive disability was significantly greater in all PD patients with fine motor skill disability than in those without disability. The results were identical for patients with PD aged (**B**) <75 or (**C**) ≥75 years. All data are presented as mean ± SD (*** *p* < 0.001).

In the non-PDD group, the disability in the two hand fine motor skills was also associated with cognitive disability. As illustrated in Figure 2, patients with fine motor skill disability also exhibited significant cognitive disability, an observation common between the subgroups of younger and older patients with PD. The significant differences persisted even after adjustments for age, sex, and H & Y stage, demonstrating that a significant association of hand fine motor skill disability with higher cognitive disability scored on a 10-point scale (OR: 1.229, 95% CI: 1.187–1.272; Supplementary Material Table S4).

## 4. Discussion

The present study revealed the association between hand fine motor skills and cognition in patients with moderate-to-advanced PD. Of the WHODAS 2.0 tasks tested, pen holding and knotting were significantly associated with the presence of dementia, with any disability in the two fine motor skills being sensitive to the presence of dementia. In addition, in moderate-to-advanced PD patients without dementia, disability in the two tasks was associated with greater cognitive disability. These results suggested that cognition influenced fine motor skill disability and that the two tasks can be employed as cognitive disability screening tools in patients with moderate-to-advanced PD.

Cognition influences fine motor skills considerably [10,11]. Although numerous studies have demonstrated that coordination and stability are primarily associated with basal ganglia, the cortical connections and functions are critical [20,21,22,23]. In a population-based longitudinal study of 2361 older patients, the time required to wear a shirt or complete a manual dexterity task was significantly longer in the preclinical phase of dementia [18]. In the Parkinsonism pathophysiology, early changes start from the medulla and pontine tegmentum and then slowly progress to the substantia nigra dopaminergic system during the mid-stage and finally to cortical deposition of Lewy bodies [24]. Braak staging systems describe the changes in the neuropathology and clinical symptoms. Hand fine motor movement performances are also associated with sensorimotor, secondary somatosensory, temporal, limbic, frontooccipital, frontoparietal, and cerebellar networks, and are well described in various studies [20,21,25]. Therefore, we deduce that the progression of Lewy deposition will be able to be observed by clinical symptoms’ deterioration, especially in intact hand motor fine skills during the baseline of diagnosis.

PD patients with mild cognitive impairment (MCI) perform significantly worse in subtests of precision dexterity and velocity of arm-hand movements than PD patients without MCI [10,18]. In addition, in a study comparing the finger tapping patterns among patients with AD, PD, and MCI and healthy older adults, higher finger tapping variability was negatively associated with cognition; moreover, patients with PD demonstrated more taps with shorter intertap intervals [17]. The presence of altered sensorimotor integration in patients with early stage PD has been reported in several electrophysiology studies [26,27]. Notably, a neuroimaging study revealed significant decreases in the volumes of occipital cortical areas in patients with PD compared with controls; these decreases were associated with decreases in the fine motor speed and set shifting (frontal lobe function). Such associations may reflect the compensatory role of the occipital cortex in primary frontostriatal pathology [13]. These correlations could explain how cognitive–sensory system interaction influences fine motor function, particularly in populations with cognitive impairment. In the present study, the performance of all three tasks (pen holding, buttoning, and knotting) investigated was worse in the PDD group than in the non-PDD group, but only pen holding and knotting remained significantly worse after adjustments for age, sex, and PD severity. Compared with the other two tasks, which could be facilitated with visual motor processing compensation, buttoning requires greater sensory cortical integration. Early defects in peripheral afferent inputs in patients with PD [28] may explain the nonsignificant differences in the buttoning performance between patients with and without cognitive impairments [29,30]. Therefore, the buttoning task may not be an appropriate progressive marker for cognition dysfunction in PD.

This study was conducted using nationwide data in Taiwan; thus, our results—confirming the cognitive–fine motor function relationship—may represent the status of most patients with moderate-to-advanced PD in Taiwan. In previous studies, the results of different screening tests have been compared, with inconsistent results reported with regard to the optimum sensitivity profile, specificity, or even time requirements [11]. Therefore, unlike common assessment tools (Mini–Mental State Examination and Montreal Cognitive Assessment), we applied the FUNDES-Adult to evaluate multiple aspects of disability. The current easiest screening tool for cognitive disability or dementia for PD patients is MMSE, which may take about 5 min [31] for each screen and is not so easy for all caregivers to evaluate. The examiners must be trained to conduct the MMSE precisely. However, hand motor disability (pen holding, buttoning, and tying a knot) can be easily assessed by caregivers, social workers, and any level of health care staff regardless of their education level and can be used as a quick screen in the outpatient department. The results indicated pen holding and knotting disability were sensitive to the presence of dementia in patients with moderate-to-advanced PD. Consequently, these two simple task tests have promising applications in outpatient clinical settings, chronic care institutions, and even in communities for the early detection of cognitive decline in patients with PD. Further education and rehabilitation for patient, families, and caregivers as an early awareness of functional decline in social relationships and life activities is crucial. Patients can be referred to physicians for further evaluation when poorer functional performances are observed. Even though many previous studies have shown a correlation between cognitive disability and hand motor skills, none have used such big samples, to the best of our knowledge, we are the first study to analyze moderate-severe PD’s motor skills and association with cognitive disability. It is suspected that for PD, a disease with impairment of motor performance, the correlation between cognitive disabilities and hand motor skills may not remain significant in other healthy elders. Our study revealed that regardless of motor disability, deterioration in PD can be detected early as a sign of cognitive involvement.

The limitations of this study include its case–control retrospective nature. We mainly focused on the PD population in Taiwan, and other ethnicities, cultures, and environments may exhibit different aspects of the disabilities examined. In addition, as aforementioned, the results could only be applied to populations with moderate-to-advanced-stage PD. The proportion of patients with PDD among the patients with moderate-to-advanced PD was 17.8% in our study population, similar to the dementia prevalence in PD reported elsewhere [32]. Second, the database we used did not contain information on the disease duration, treatment status, or comorbidities. For instance, comorbidities of cerebrovascular disease, mood disorders, drug exposures, motor subtype, or specific PD symptoms (i.e., motor fluctuation and complications) may have been confounding factors in the present study. Third, the lack of UPDRS score data during WHODAS assessment is also one of our limitations. However, in the present study, the patients were enrolled based on the H & Y stage, which were categorized as the moderate to severe PD group patients. In the statistical analysis (Figure 1), the severity of PD, based on the H & Y stage, was one of the adjusting factors to minimize the impact of motor dysfunction on the association. Fourth, PD and dementia diagnoses may vary across different health care institutions; therefore, reviewers may misdiagnosis patients as having diseases not relevant to this study. Nevertheless, we included only patients who received disability certification for PD from board-certified neurologists, thus minimizing the misdiagnosis risks. 

## 5. Conclusions

Cognitive status was strongly associated with hand fine motor skills in patients with moderate-to-advanced PD, particularly pen holding and knotting. In the future, these simple tasks may have promising applications as cost-effective screening tests for the early detection of cognitive dysfunction in patients with PD. Further studies exploring specific hand fine movement performance in PD patients with MCI and PDD and investigating the underlying neurophysiological circuits and mechanisms are warranted.

## Figures and Tables

**Table 1:** Patient demographic data.

	PDD (*n* = 612)	Non-PDD (*n* = 2828)	*p* Value
Female (*n*, %)	326 (53.3)	1,380 (48.8)	0.04
Age (y/o, mean [SD])	77.1(7.3)	73.1(8.9)	<0.001
Education > 6 years (*n*, %)	67 (21.1)	573 (20.3)	N.S.
Hoehn-Yahr Stage (*n*, %)			<0.001
Stage 3	212 (34.6)	1,146 (40.5)	
Stage 4	252 (41.2)	1,197 (42.3)	
Stage 5	148 (24.2)	485 (17.2)	
World Health Organization Disability Assessment 2.0 (mean [SD])	55.1(22.2)	49.5 (21.4)	<0.001
Hand Fine Motor Skill (*n*, %)			
Pen-holding	1.2 (1.5)	0.8 (1.3)	<0.001
Buttoning	2.0 (1.6)	1.7 (1.5)	<0.001
Knotting	2.0 (1.6)	1.6 (1.5)	<0.001

Parkinson’s disease dementia (PDD), non-Parkinson’s disease dementia (non-PDD).

**Table 2:** Distribution of hand fine motor skill disabilities among patients with and without Parkinson’s disease-related dementia.

	PDD	Non-PDD
Disability	445	1884
No Disability	167	944

Parkinson’s disease dementia (PDD), non-Parkinson’s disease dementia (non-PDD).

## Supplementary Materials

The following are available online at https://www.mdpi.com/2076-3425/10/6/337/s1, Figure S1: Patient selection process; Table S1: Items of the eight domains of the Functioning Scale of the Disability Evaluation System-Adult Version; Table S2: Multivariate logistic regression models for the association between the different hand fine motor skills and presence of dementia after adjustments for age, sex, and the Hoehn and Yahr stage of Parkinson’s disease, Table S3: Distribution of hand fine motor skill disabilities among patient age groups; Table S4: Multivariate logistic regression models for association between hand fine motor skill disabilities and cognitive disability after adjustments for age, sex, and the Hoehn and Yahr stage of Parkinson’s disease.
