# Supplementary material for: Hand Fine Motor Skill Disability Correlates with Cognition in Patients with Moderate-to-Advanced Parkinson’s Disease
Source: Brain Sci. 2020 Jun 2;10(6):337. doi: 10.3390/brainsci10060337 (PMC7349665; doi:10.3390/brainsci10060337)
Supplement: Supplementary file 1 [file brainsci-10-00337-s001.pdf]

brain sciences

MDPI

Supplementary Materials: Hand Fine Motor Skill Disability Correlates with Cognition in Patients with Moderate-to-Advanced Parkinson's Disease

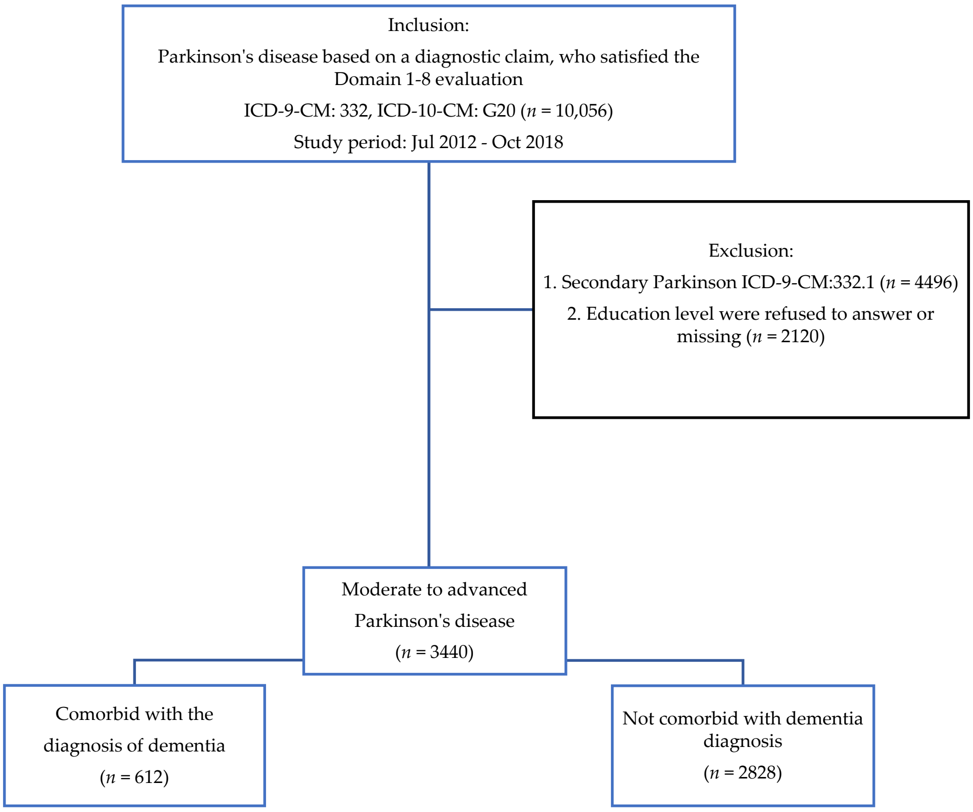

Figure S1. Patient selection process

Table S1. Items of the eight domains of the Functioning Scale of the Disability Evaluation System-Adult Version.

Domains	Items and labels
Do1 Cognition	D1.1 Concentration
(6 items) [a]	D1.2 Remembering to do important things
	D1.3 Problem-solving
	D1.4 Learning a new task
	D1.5 Understanding
	D1.6 Conversation
Do2 Mobility	D2.1 Standing for long periods
(5 items) [a]	D2.2 Standing up from sitting
	D2.3 Moving around inside home
	D2.4 Getting out of home
Do3 Self-care	D3.1 Washing whole body
(4 items) [a]	D3.2 Getting dressed
	D3.3 Eating
	D3.4 Staying by self for a few days

Do4 Getting along (5 items)[a]	D4.1 Dealing with strangers<br>D4.2 Maintaining a friendship<br>D4.3 Getting along with people close to<br>D4.4 Making new friends<br>D4.5 Sexual activities
Do5 Life activities Do5.1 Household activities (4 items) [a]	D5.1 Household responsibilities<br>D5.2 Do important household tasks well<br>D5.3 Do all needed household work<br>D5.4 Household work performed as quickly as needed
Do5.2 Work or school activities (4 items) [a]	D5.5 Day-to-day work/school<br>D5.6 Do important work/school tasks well<br>D5.7 Getting done all needed work<br>D5.8 Work performed as quickly as needed
Do6 Participation (8 items)[a]	D6.1 Joining in community activities<br>D6.2 Because of environmental barriers<br>D6.3 Others affects one's dignity<br>D6.4 Health affects time consumption<br>D6.5 Health affects one's emotion<br>D6.6 Health affects family finances<br>D6.7 Health affects family Not linkable (impact question)<br>D6.8 Doing things for relaxation or pleasure
Do7 Environmental attributes (8 items)[c]	D7.1 Food and drugs for personal consumption<br>D7.2 Products and technology for personal use in daily living<br>D7.3 Products and technology for personal indoor and outdoor mobility and transportation<br>D7.4 Products and technology for communication<br>D7.5 Products and technology for education<br>D7.6 Financial assets<br>D7.7 Climate<br>D7.8 Social security services
Do8 Motor action (7 items)[b]	D8.1 Pick up a pen<br>D8.2 Button up<br>D8.3 Tie something<br>D8.4 Stand up from chair sitting<br>D8.5 Bend down to pick something up<br>D8.6 Walk for 3 m and return<br>D8.7 Sit onto the chair

One extra item is used to ask about having assistive devices or personal assistance in daily life in Domains 1-6 and Domain 8. Two extra items are used to ask about the health impacts on working and salary in each dimension of Domain 6; a, Each item has performance and capability dimensions through interviewing, scoring 0e4 for level of difficulty (rated as follows: 0 = no difficulty; 1 = slight; 2=medium; 3 = high; 4 = extremely high or unable and 9 for not applicable.); b, Each item has a capability dimension through interviewing, scoring 0-4 for degree of personal assistance with usual devices, and each item has a capacity dimension through direct testing without mobility devices, scoring 0-4 (0 = no assistance; 1 =supervision or verbal cue; 2 = a little physical assistance; 3 = lots of physical assistance; 4 = complete physical assistance); c, Through interviewing, dichotomy score: 0 = no environmental barrier or open access; 1 = encounter environmental barrier.

Table S2. Multivariate logistic regression models for the association between the different hand fine motor skills and presence of dementia after adjustments for age, sex, and the Hoehn and Yahr stage of Parkinson's disease.

	β	Odds ratio	95% Confidence index		p value
Pen-holding	0.125	1.133	1.055	1.217	0.001
Buttoning	0.040	1.041	0.974	1.112	0.235
Knotting	0.095	1.099	1.030	1.173	0.004

Table S3. Distribution of hand fine motor skill disabilities among patient age groups.

(A) PD patients less than 75 years old.

	PDD	Non-PDD
Disability	117	884
No Disability	66	565

Sensitivity of detecting dementia by the presence of disability with had fine motor skills = 63.9%; Abbreviation: Parkinson's disease dementia (PDD), non- Parkinson's disease dementia (non-PDD)

(B) PD patients 75 years old and above.

	PDD	Non-PDD
Disability	328	1000
No Disability	101	379

Sensitivity of detecting dementia by the presence of disability with had fine motor skills =76.5%; Abbreviation: Parkinson's disease dementia (PDD), non- Parkinson's disease dementia (non-PDD)

Table S4. Multivariate logistic regression models for association between hand fine motor skill disabilities and cognitive disability after adjustments for age, sex, and the Hoehn and Yahr stage of Parkinson's disease.

	**β**	**Odds Ratio**	**95% Confidence Index**		***p* Value**
Intercept	−0.455				<0.001
Gender(ref = Male)					
Female	−0.214	0.807	0.683	0.955	0.012
Age (ref ≤ 75)					
75 years & above	0.187	1.206	1.015	1.433	0.034
Hoehn-Yahr Stage (ref = Stage 3)					
Stage 4	0.422	1.524	1.277	1.820	<0.001
Stage 5	1.230	3.420	2.472	4.733	<0.001
Cognition	0.206	1.229	1.187	1.272	<0.001

Cognitive disability was classified on a 10-point scale.
